# Assessment of the Diagnostic Performance of Endoscopic Ultrasonography After Conventional Endoscopy for the Evaluation of Esophageal Squamous Cell Carcinoma Invasion Depth

**DOI:** 10.1001/jamanetworkopen.2021.25317

**Published:** 2021-09-15

**Authors:** Ryu Ishihara, Junki Mizusawa, Ryoji Kushima, Noriko Matsuura, Tomonori Yano, Tomoko Kataoka, Haruhiko Fukuda, Noboru Hanaoka, Toshiyuki Yoshio, Seiichiro Abe, Yoshinobu Yamamoto, Shinji Nagata, Hiroyuki Ono, Masashi Tamaoki, Naohiro Yoshida, Kohei Takizawa, Manabu Muto

**Affiliations:** 1 Department of Gastrointestinal Oncology, Osaka International Cancer Institute, Osaka, Japan; 2 Japan Clinical Oncology Group Data Center/Operations Office, National Cancer Center Hospital, Tokyo, Japan; 3 Department of Clinical Laboratory Medicine (Diagnostic Pathology), Shiga University of Medical Science, Japan; 4 Division of Research and Development for Minimally Invasive Treatment, Cancer Center, Keio University School of Medicine, Tokyo, Japan; 5 Department of Gastroenterology and Endoscopy, National Cancer Center Hospital East, Kashiwa, Japan; 6 Department of Gastroenterology, Osaka Red Cross Hospital, Osaka, Japan; 7 Department of Gastroenterology, Cancer Institute Hospital, Japanese Foundation for Cancer Research, Tokyo, Japan; 8 Endoscopy Division, National Cancer Center Hospital, Tokyo, Japan; 9 Department of Gastroenterological Oncology, Hyogo Cancer Center, Akashi, Japan; 10 Department of Gastroenterology, Hiroshima City Asa Citizens Hospital, Hiroshima, Japan; 11 Division of Endoscopy, Shizuoka Cancer Center, Shizuoka, Japan; 12 Department of Therapeutic Oncology, Kyoto University Graduate School of Medicine, Kyoto, Japan; 13 Department of Gastroenterology, Ishikawa Prefectural Central Hospital, Kanazawa, Japan

## Abstract

**Importance:**

Distinguishing between mucosal and submucosal cancers is important for selecting the optimal treatment for patients with esophageal squamous cell carcinoma (ESCC); however, standard procedures for diagnosing cancer invasion depth have not yet been determined.

**Objective:**

To evaluate the diagnostic performance of endoscopic ultrasonography (EUS) after conventional endoscopy for the evaluation of ESCC invasion depth.

**Design, Setting, and Participants:**

This prospective single-arm confirmatory diagnostic study comprising 372 patients with T1 esophageal cancer was conducted at 41 secondary or tertiary hospitals in Japan. Enrollment began on July 20, 2017; patients were enrolled in 2 steps, with the first registration occurring from August 4, 2017, to December 11, 2019, and the second from August 9, 2017, to December 11, 2019. After the completion of all first and second registration examinations, patients received treatment and were followed up for 30 days, with follow-up ending on February 14, 2020. Patients were eligible for inclusion if they had pathologically or endoscopically diagnosed esophageal cancer with T1 clinical depth of invasion.

**Interventions:**

In the first registration, nonmagnifying endoscopy (non-ME) and magnifying endoscopy (ME) were used to diagnose cancer invasion depth. In the second registration, patients from the first registration who had cancers invading the muscularis mucosa or submucosa were enrolled and received EUS. After completion of the protocol examinations, patients received treatment with endoscopic resection or esophagectomy. The pathological results of the resected specimens were used as the reference standard for evaluating cancer invasion depth.

**Main Outcomes and Measures:**

The primary end point was the proportion of overdiagnosis of submucosal cancer (defined as invasion depth >200 μm) after receipt of non-ME and ME, with or without the addition of EUS. The secondary end points were underdiagnosis, sensitivity, and specificity.

**Results:**

Among 372 patients enrolled in the first registration, 371 received non-ME and ME. Of those, 300 patients were enrolled in the second registration, and 293 patients received EUS. A total of 269 patients (217 men [80.7%]; median age, 69 years; interquartile range, 62-75 years) were included in the final analysis. The addition of EUS was associated with a 6.6% increase in the proportion of overdiagnosis (from 16 of 74 patients [21.6%; 95% CI, 12.9%-32.7%] after non-ME and ME to 29 of 103 patients [28.2%; 95% CI, 19.7%-37.9%] after the addition of EUS; 1-sided *P* = .93). All subgroup analyses found similar increases in overdiagnosis of submucosal cancer. The addition of EUS was associated with a 4.5% reduction in the proportion of underdiagnosis (from 57 of 195 patients [29.2%; 95% CI, 23.0%-36.2%] after non-ME and ME to 41 of 166 patients [24.7%; 95% CI, 18.3%-32.0%] after the addition of EUS). After non-ME and ME, diagnostic sensitivity was 50.4% (95% CI, 41.0%-59.9%), specificity was 89.6% (95% CI, 83.7%-93.9%), and accuracy was 72.9% (95% CI, 67.1%-78.1%). After the addition of EUS, diagnostic sensitivity was 64.3% (95% CI, 54.9%-73.1%), specificity was 81.2% (95% CI, 74.1%-87.0%), and accuracy was 74.0% (95% CI, 68.3%-79.1%).

**Conclusions and Relevance:**

This study found that the addition of EUS was not associated with improvements in the diagnostic accuracy of cancer invasion depth. These findings do not support the routine use of EUS after conventional endoscopy for evaluating the invasion depth among patients with T1 ESCC.

## Key Points

**Question:**

Is the performance of endoscopic ultrasonography after conventional endoscopy associated with improvements in diagnosing the invasion depth of esophageal squamous cell carcinoma?

**Findings:**

In this diagnostic study of 372 patients with esophageal cancer, the performance of endoscopic ultrasonography after nonmagnifying and magnifying endoscopy was not associated with improvements in diagnostic performance for differentiating between submucosal and mucosal cancers in terms of overdiagnosis and accuracy.

**Meaning:**

This study suggests that endoscopic ultrasonography after nonmagnifying and magnifying endoscopy is not helpful for evaluating the invasion depth of T1 esophageal squamous cell carcinoma.

## Teaser

This study assesses the performance of endoscopic ultrasonography after nonmagnifying and magnifying endoscopy for the diagnosis of cancer invasion depth among patients with esophageal squamous cell carcinoma.

## Introduction

Esophageal cancer is the seventh most common cancer and the sixth most common reason for cancer-associated mortality worldwide.^1^ Endoscopic resection is a minimally invasive treatment option for early-stage esophageal squamous cell carcinoma (ESCC).^2,3,4,5^ Given that endoscopic resection can be curative for mucosal (T1a) cancers,^3,6,7^ it is important to differentiate between T1a (mucosal) and T1b (submucosal) cancers. However, a clinical diagnosis of microinvasion into the submucosa is challenging, and T1b cancer that has invaded the submucosa by 200 μm or less (ie, submucosal 1 cancer) is clinically categorized together with muscularis mucosal cancer (ie, muscularis mucosal/submucosal 1 cancer).^8,9,10^ Guidelines recommend differentiating between invasion depths of 200 μm or less (mucosal/submucosal 1 cancer) and greater than 200 μm (submucosal 2 cancer) to allow the appropriate treatment selection for patients with ESCC.^6,11^

In general, endoscopic evaluation using nonmagnifying endoscopy (non-ME) followed by magnifying endoscopy (ME) is the most common initial procedure in Japan for differentiating between muscularis mucosal/submucosal 1 and submucosal 2 cancers.^12^ In contrast, endoscopic ultrasonography (EUS) is not used as a standard procedure because of conflicting results regarding its diagnostic performance.^13,14^ The use of EUS is recommended for staging T1 esophageal cancer in some guidelines^7,15,16^ and by some experts^17^ but is not recommended in other guidelines.^6,18^ It is therefore necessary to evaluate the diagnostic performance of EUS after non-ME and ME to optimize the diagnostic process for cancer invasion depth.

Previous reports on the performance of non-ME, ME, and EUS have been limited by their retrospective and single-center designs or small samples.^10,19,20,21,22,23,24^ A systematic review of these studies^12^ found a high risk of bias regarding patient selection and study flow. We conducted a multicenter prospective study to evaluate the additional diagnostic value of performing EUS after non-ME and ME by evaluating the relative incidence of overdiagnosis and underdiagnosis in patients with clinical T1 ESCC.

## Methods

This single-arm prospective confirmatory study was conducted in 41 secondary or tertiary care hospitals across Japan. The study protocol was approved by the ethics committees of each participating hospital, and written informed consent was obtained from all patients. This study was conducted in accordance with the Declaration of Helsinki^25^ and followed the Standards for Reporting of Diagnostic Accuracy (STARD) reporting guideline for diagnostic accuracy studies.

### Eligibility Criteria

Study enrollment began on July 20, 2017, and patients were enrolled in 2 steps. Eligibility for the first registration (conducted from August 4, 2017, to December 11, 2019) was evaluated based on endoscopic examination at the time of esophageal cancer detection. Patients were then asked to undergo endoscopic examination with non-ME and ME to diagnose cancer invasion depth within 14 days of the first registration; the endoscopic results were used to assess their eligibility for the second registration. Patients enrolled in the second registration (conducted from August 9, 2017, to December 11, 2019) received EUS. The main inclusion criteria for the first registration were (1) pathologically confirmed ESCC based on criteria in the *Japanese Classification of Esophageal Cancer*, 11th edition,^8^ or endoscopically diagnosed ESCC; (2) clinically diagnosed cN0M0 cancer if computed tomography was conducted; (3) cancer located in the thoracic esophagus; (4) cancer invasion depth of cT1 diagnosed by non-ME; (5) age 20 to 85 years; (6) Eastern Cooperative Oncology Group performance status of 0 to 2; (7) no history of thoracic radiotherapy or esophagectomy; and (8) no request to receive chemoradiotherapy as first-line treatment.

The main inclusion criteria for the second registration were (1) cancer invasion depth of muscularis mucosa/submucosa diagnosed by non-ME and ME and (2) major axis length of 50 mm or less. The main exclusion criteria were (1) clinical epithelial/lamina propria cancer diagnosed by non-ME; (2) active infection requiring systemic therapy; (3) pregnant or breastfeeding; and (4) psychiatric diseases.

### Study Design

**Figure 1.**
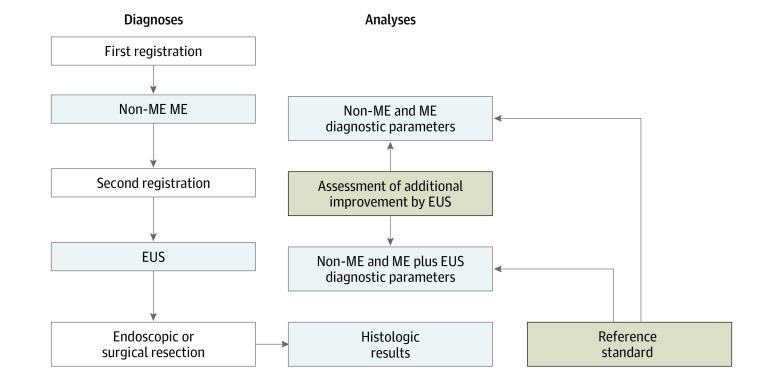
Diagnoses and Analyses EUS indicates endoscopic ultrasonography; ME, magnifying endoscopy; and non-ME, nonmagnifying endoscopy.

After the first registration (Figure 1), protocol examinations for the diagnosis of cancer invasion depth were conducted using non-ME with white light imaging and ME with narrow-band or blue laser imaging (GIF-Q240Z, GIF-H260Z, GIF-FQ260Z, or GIF-H290Z [Olympus] and EG-L590ZW or EG-L600ZW [Fujifilm]). Cancer invasion depth diagnoses were categorized as clinical epithelium/lamina propria, clinical muscularis mucosa/submucosa 1, or clinical submucosa 2 or deeper.

After the second registration, patients diagnosed with clinical muscularis mucosa/submucosa 1 or clinical submucosa 2 who fulfilled the other inclusion criteria received EUS. The examiners were informed of the non-ME and ME results before performing EUS. Endoscopic ultrasonography was conducted using a 20 to 30 MHz miniature probe, and scanning was performed using the water-filling, jelly-filling, or water-filled balloon method. According to the protocol, non-ME and ME were performed within 2 weeks after the first registration, and EUS was performed within 2 weeks after the second registration. To ensure study quality, all protocol examinations were performed by certified endoscopists (including some authors: R.I., N.M., T.Y., N.H., T.Y., S.A., Y.Y., S.N., H.O., M.T., N.Y., K.Y., and M.M.) who had performed 20 or more non-ME and ME examinations and 10 or more EUS examinations of ESCC cases.

After completion of the protocol diagnostic procedures, patients received treatment for ESCC. Endoscopic resection and esophagectomy were the main treatments administered because patients who initially wanted to receive chemoradiotherapy were excluded before the first registration. Treatment with endoscopic resection or esophagectomy was selected based on cancer invasion depth, lesion size, and the patient’s condition and choice. According to the protocol, treatment was delivered within 8 weeks after EUS because cancer invasion may have progressed if a longer interval were used. Patients were followed up for 30 days after the completion of endoscopic examinations and treatments and were assessed for adverse events according to criteria in the *Common Terminology Criteria for Adverse Events*, version 4.0.^26^ The last date of follow-up was February 14, 2020.

### Classification Used for Diagnosis

The typical endoscopic appearances of superficial ESCC by non-ME with white light imaging^9^ were classified as (1) clinical epithelium/lamina propria, indicating flat lesions without protrusion or depression; (2) clinical muscularis mucosa/submucosa 1, indicating flat lesions with uneven surfaces and protrusions of less than 1 mm or shallow depression; and (3) clinical submucosa 2, indicating lesions with protrusions of 1 mm or more or deep depression. The typical findings of ME for each tumor category^10^ were classified as (1) clinical epithelium/lamina propria, indicating looplike abnormal vessels showing dilated, tortuous, irregular caliber, and heterogeneous shape; (2) clinical muscularis mucosa/submucosa 1, indicating abnormal blood vessels (multiple and dendritic) that were poorly looped; and (3) clinical submucosa 2, indicating highly dilated irregular vessels that were poorly looped.

The performance of EUS using a miniature probe usually depicts the normal esophageal wall as a 9-layered structure. The typical findings of EUS for each tumor category^16^ were classified as (1) clinical epithelium/lamina propria, indicating no interruption in the third layer; (2) clinical muscularis mucosa/submucosa 1, indicating interruption of the third layer and no tumor echo in the fourth layer; and (3) clinical submucosa 2, indicating tumor echo in the fourth layer. The level of confidence of the endoscopic ultrasonographic diagnosis was classified as high (ie, an endoscopic finding with high reproducibility) or low (ie, an endoscopic finding with low reproducibility).

### Diagnostic Procedure

The diagnostic procedure was designed to simulate our daily practice. Cancer invasion depth was initially diagnosed using non-ME with white light imaging followed by ME with narrow-band or blue laser imaging. The non-ME and ME diagnosis was based on comprehensive results from both endoscopies using the most typical and reliable findings. The performance of non-ME and ME was followed by EUS. Diagnosis after the addition of EUS was also based on comprehensive results from all examinations using the most typical and reliable findings.

### Pathological Examinations

Pathological examinations were performed according to the standard methods proposed by the *Japanese Classification of Esophageal Cancer*, 11th edition.^8^ After fixation, all resected specimens were cut into 2-mm-wide longitudinal slices for endoscopic resection and as thinly as possible but no thinner than 3-mm-wide longitudinal slices for esophagectomy. The tissue specimens were embedded in paraffin, sectioned at 4 μm, and stained with hematoxylin and eosin, desmin, monoclonal antibody D2-40, and elastic fiber.

Cancer invasion depth was divided into the following categories: (1) pathological epithelium, (2) pathological lamina propria, (3) pathological muscularis mucosa, (4) pathological submucosa 1, and (5) pathological submucosa 2. The histologic type and cancer invasion depth were diagnosed through central review by 3 expert pathologists (one of whom was R.K.) and were used as the reference standards (Figure 1).

### Statistical Analysis

This study was designed to confirm substantial improvement in diagnostic accuracy for the performance of EUS after non-ME and ME. The primary end point for the assessment of diagnostic accuracy was defined as the proportion of patients with overdiagnosis of submucosal 2 cancer, and the main secondary end point was defined as the proportion of patients with underdiagnosis of submucosal 2 cancer. Considering the patient burden associated with a misdiagnosis of cancer invasion depth, overdiagnosis was considered to be more clinically disadvantageous than underdiagnosis and was therefore selected as the primary end point.

Other secondary end points included diagnostic sensitivity, specificity, positive predictive value, and negative predictive value for submucosal 2 cancer and adverse events. The proportion of patients with overdiagnosis was defined as the proportion with pathological submucosal 1 or shallower lesions that were diagnosed as clinical submucosal 2 or deeper lesions. The proportion of patients with underdiagnosis was defined as the proportion of patients with pathological submucosal 2 or deeper lesions that were diagnosed as clinical submucosal 1 or shallower lesions.

The proportions of overdiagnosis and underdiagnosis were used as the primary and most important secondary end points because of their relevance to decisions that may result in overtreatment or undertreatment, respectively. Based on our tentative calculations, a substantial improvement in overdiagnosis would be confirmed by a 7% decrease in the proportion of overdiagnosis after the addition of EUS. We prespecified that the usefulness of EUS would be confirmed by a substantial improvement in the proportion of overdiagnosis and a 7% or lower decrease in the proportion of underdiagnosis.

Based on previous studies, we assumed that the proportion of patients with pathological submucosal 2 cancer was 40%, the proportion of overdiagnosis after receipt of non-ME and ME was 28%, and the proportion of overdiagnosis after receipt of non-ME and ME plus EUS was 19%.^4,24^ According to Wang et al,^27^ we required a sample of 264 patients with a power of 80%, a 1-sided α of 5%, a sensitivity of 90% for EUS, and a correlation between the 2 tests of 0.3. The required sample for the second registration was thus initially set at 264 patients but later revised to 300 patients given that some patients would likely be excluded from the final analysis.

The primary analysis for comparing non-ME and ME with non-ME and ME plus EUS was performed using generalized estimating equations with a logit link function, a binomial variance distribution, and an independent working correlation structure.^28^ Prespecified subgroup analyses were conducted according to macroscopic type (with or without an elevated component) and confidence in endoscopic ultrasonographic diagnosis (low or high). Post hoc subgroup analyses were conducted according to tumor size (≤2 cm or >2 cm) and location (upper, middle, or lower thoracic esophagus). The results of these subgroup analyses were considered to be exploratory in nature. The Japan Clinical Oncology Group Data Center was responsible for data management, central monitoring, and statistical analyses. All statistical analyses were performed using SAS software, version 9.4 (SAS Institute).

## Results

**Figure 2.**
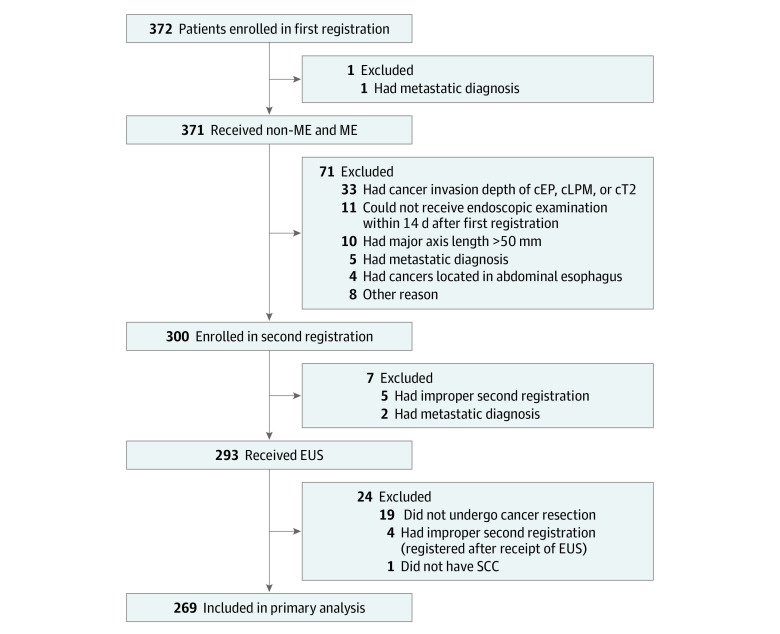
Flowchart of Patients cEP indicates clinical epithelium; cLPM, clinical lamina propria; EUS, endoscopic ultrasonography; ME, magnifying endoscopy; non-ME, nonmagnifying endoscopy; and SCC, squamous cell carcinoma.

A total of 372 patients were enrolled in the first registration, and 371 patients received non-ME and ME (Figure 2). Of those, 300 patients were enrolled in the second registration, and 293 patients received EUS. In total, 269 patients (217 men [80.7%]; median age, 69 years; interquartile range [IQR], 62-75 years) were included in the primary analyses (Table). The reasons for excluding 72 of 372 patients (19.4%) from the second registration and 31 of 300 patients (10.3%) from the final analysis are shown in Figure 2 and eTable 1 in the Supplement. The median time from EUS to endoscopic resection and esophagectomy was 18 days (IQR, 10-27 days) and 39 days (IQR, 28-57 days), respectively.

**Table.** Characteristics of Patients and Cancers Included in Primary Analysis

Characteristic	Patients, No. (%)
Patients, No.	269
Age, median (IQR), y	69 (62-75)
Sex	
Male	217 (80.7)
Female	52 (19.3)
ECOG performance status	
0	262 (97.4)
1	6 (2.2)
2	1 (0.4)
Tumor location	
Upper thoracic esophagus	41 (15.2)
Middle thoracic esophagus	153 (56.9)
Lower thoracic esophagus	75 (27.9)
Macroscopic type^a^	
0-I	11 (4.1)
0-IIa	22 (8.2)
0-IIb	6 (2.2)
0-IIc	121 (45.0)
Mixed	
Including 0-I	42 (15.6)
Not including 0-I	67 (24.9)
Tumor diameter, median (IQR), cm	2.0 (1.5-3.0)
Tumor circumference	
<1/4	95 (35.3)
≥1/4 to <1/2	95 (35.3)
≥1/2 to <3/4	56 (20.8)
≥3/4	23 (8.6)
Procedure time of non-ME and ME, median (IQR), min	8 (6-11)
Diagnoses by non-ME and ME	
Clinical MM/SM1	195 (72.5)
Clinical SM2	74 (27.5)
Procedure time of EUS, median (IQR), min	9 (6-13)
Scanning method of EUS	
Water–filling	131 (48.7)
Jelly–filling	118 (43.9)
Water–filled balloon	20 (7.4)
Diagnoses by non-ME and ME plus EUS	
Clinical EP/LPM	22 (8.2)
Clinical MM/SM1	144 (53.5)
≤Clinical SM2	103 (38.3)
Confidence level of EUS diagnoses	
Low	109 (40.5)
High	160 (59.5)
Resection	
Endoscopic	200 (74.3)
Surgical	69 (25.7)

Abbreviations: ECOG, Eastern Cooperative Oncology Group; EP, epithelium; EUS, endoscopic ultrasonography; IQR, interquartile range; LPM, lamina propria; ME, magnifying endoscopy; MM, muscularis mucosa; non–ME, nonmagnifying endoscopy; SM, submucosa.

^a^ Type 0-I indicates protruded lesion; type 0-IIa, superficial and elevated lesion; type 0-IIb, flat lesion; and type 0-IIIc, superficial and depressed lesion.

**Figure 3.**
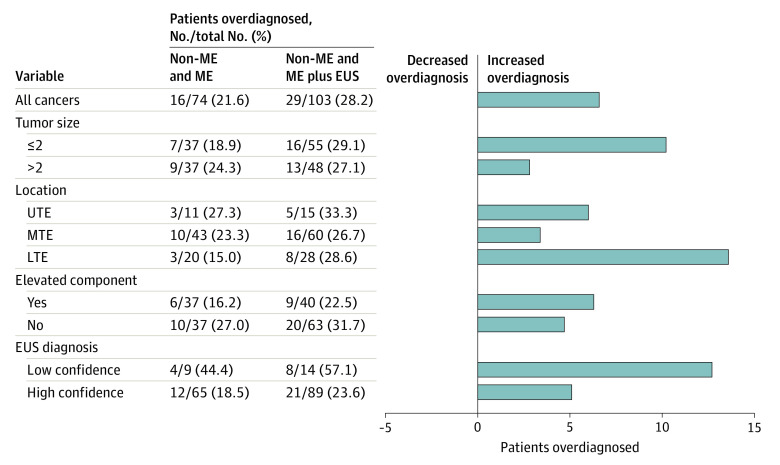
Proportion of Overdiagnosis Before and After Endoscopic Ultrasonography EUS indicates endoscopic ultrasonography; LTE, lower thoracic esophagus; ME, magnifying endoscopy; MTE, middle thoracic esophagus; non-ME, nonmagnifying endoscopy; and UTE, upper thoracic esophagus.

**Figure 4.**
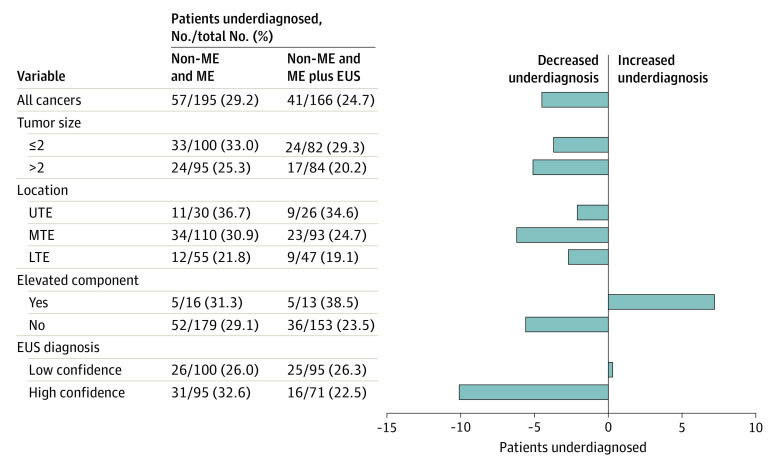
Proportion of Underdiagnosis Before and After Endoscopic Ultrasonography EUS indicates endoscopic ultrasonography; LTE, lower thoracic esophagus; ME, magnifying endoscopy; MTE, middle thoracic esophagus; non-ME, nonmagnifying endoscopy; and UTE, upper thoracic esophagus.

After the addition of EUS, the number of patients diagnosed with clinical submucosal 2 or deeper cancer increased from 74 to 103, and the proportion of overdiagnosis increased by 6.6% (from 16 of 74 patients [21.6%; 95% CI, 12.9%-32.7%] after non-ME and ME to 29 of 103 patients [28.2%; 95% CI, 19.7%-37.9%] after the addition of EUS; 1-sided *P* = .93). Subgroup analyses found similar increases in overdiagnosis across all subgroups (eg, among patients with tumors ≤2 cm vs >2 cm, the proportion of overdiagnosis was 7 of 37 patients [18.9%] vs 9 of 37 patients [24.3%], respectively, after non-ME and ME and 16 of 55 patients [29.1%] vs 13 of 48 patients [27.1%] after the addition of EUS) (Figure 3). The proportion of underdiagnosis decreased by 4.5% after EUS (from 57 of 195 patients [29.2%; 95% CI, 23.0%-36.2%] after non-ME and ME to 41 of 166 patients [24.7%; 95% CI, 18.3%-32.0%] after the addition of EUS) (Figure 4).

The diagnostic sensitivity, specificity, positive predictive value, and negative predictive value for submucosal 2 or deeper cancer are shown in eTable 2 in the Supplement. The addition of EUS improved diagnostic sensitivity (from 58 of 115 patients [50.4%; 95% CI, 41.0%-59.9%] after non-ME and ME to 74 of 115 patients [64.3%; 95% CI, 54.9%-73.1%] after the addition of EUS) and negative predictive value (from 138 of 195 patients [70.8%; 95% CI, 63.8%-77.0%] after non-ME and ME to 125 of 166 patients [75.3%; 95% CI, 68.0%-81.7%] after the addition of EUS), but specificity (from 138 of 154 patients [89.6%; 95% CI, 83.7%-93.9%] after non-ME and ME to 125 of 154 patients [81.2%; 95% CI, 74.1%-87.0%] after the addition of EUS) and positive predictive value (from 58 of 74 patients [78.4%; 95% CI, 67.3%-87.1%] after non-ME and ME to 74 of 103 patients [71.8%; 95% CI, 62.1%-80.3%] after the addition of EUS) decreased, and no substantial improvement in diagnostic accuracy was observed (from 196 of 269 patients [72.9%; 95% CI, 67.1%-78.1%] after non-ME and ME to 199 of 269 patients [74.0%; 95% CI, 68.3%-79.1%] after the addition of EUS). The diagnostic accuracy of non-ME alone was 199 of 269 patients (74.0%; 95% CI, 68.3%-79.1%), representing a potential misdiagnosis of cancer invasion depth in 70 patients (26.0%) compared with 73 patients (27.1%) who received non-ME and ME and 70 patients (26.0%) who received additional EUS.

The clinical diagnoses after non-ME and ME with or without the addition of EUS as well as treatments and pathological diagnoses of the resected specimens are shown in the eFigure in the Supplement. Six of 166 patients (3.6%) diagnosed with clinical submucosal 1 or shallower cancer received esophagectomy compared with 63 of 103 patients (61.2%) diagnosed with clinical submucosal 2 or deeper cancer. Of the 69 patients who received esophagectomy, 17 patients (24.6%) had pathological submucosal 1 or shallower cancer, which could potentially be cured by endoscopic resection.

In the subgroup analyses according to non-ME and ME diagnosis (eFigure in the Supplement), among 74 patients diagnosed with clinical submucosal 2 or deeper cancer by non-ME and ME, the addition of EUS corrected an overdiagnosis in 5 patients (6.8%) and produced an underdiagnosis in 2 patients (2.7%). Among 195 patients diagnosed with clinical submucosal 1 or shallower cancer by non-ME and ME, the addition of EUS corrected an underdiagnosis in 18 patients (9.2%) and produced an overdiagnosis in 18 patients (9.2%).

Adverse events were analyzed in 371 patients who received non-ME and ME and 293 patients who received EUS. Grade 1 hypotension occurred in 1 patient (0.3%), grade 2 atrial fibrillation in 1 patient (0.3%), and grade 2 hypoxia in 2 patients (0.5%) during non-ME and ME. Grade 2 atrial fibrillation occurred in 1 patient (0.3%) and grade 2 hypoxia in 2 patients (0.7%) during EUS. Grade 1 respiratory, thoracic, and mediastinal disorders occurred in 1 patient (0.3%) during follow-up after EUS.

## Discussion

To our knowledge, this diagnostic study is the first multicenter prospective investigation to evaluate the performance of EUS for diagnosing cancer invasion depth in patients with ESCC. The addition of EUS was associated with a 6.6% increase in the proportion of overdiagnosis and a 4.5% decrease in the proportion of underdiagnosis. Overdiagnosis of invasion depth means that cancers that are potentially curable through endoscopic resection may be treated with esophagectomy, whereas underdiagnosis of invasion depth means that cancers may be treated with endoscopic resection, with no curative benefit. An increase in overdiagnosis is considered to have a greater impact than an increase in underdiagnosis because patients with overdiagnosis may receive unnecessary esophagectomy, which is more invasive than unnecessary endoscopic resection performed because of underdiagnosis. Considering the risk-benefit balance of adding EUS after conventional endoscopy, the current results suggest that the routine use of EUS is not beneficial for patients with T1 ESCC. However, the findings do not negate the utility of EUS for the diagnosis of deeper cancers or lymph node metastasis.

In addition to overdiagnoses and underdiagnoses, we also evaluated diagnostic parameters, such as accuracy, sensitivity, and specificity. Of these parameters, accuracy has been the most widely used in previous studies because it integrates sensitivity and specificity. In this study, the clinical benefit of adding EUS was not confirmed with regard to accuracy.

The number of patients diagnosed with clinical submucosal 2 or deeper cancer increased from 74 to 103 after the addition of EUS, and the number of patients overdiagnosed with clinical submucosal 2 or deeper cancer increased from 16 to 29 patients after EUS. These results suggest that EUS may produce an overdiagnosis of cancer invasion depth. Diagnosis of clinical submucosal 2 cancer by EUS is usually based on the existence of a low-echoic lesion in the submucosa. However, esophageal glands, ducts, and vessels can also appear as low-echoic lesions in the submucosa, potentially producing misdiagnosis of cancer invasion depth by EUS. These factors could account for the overdiagnosis of clinical submucosal 2 or deeper cancer after the addition of EUS.

The addition of EUS was associated with increases in the proportion of overdiagnosis in most subgroups, and this disadvantage did not appear to be balanced by a decrease in underdiagnosis. However, the decrease in overdiagnosis was more obvious than the increase in underdiagnosis in the subgroup of patients diagnosed with clinical submucosal 2 or deeper cancer by non-ME and ME. A 6.8% decrease in overdiagnosis in this subgroup represents a modest proportion given that we had considered a decrease of 7% to indicate substantial improvement. Although the addition of EUS may generally be associated with an overdiagnosis of cancer invasion depth, its use may be beneficial in patients with clinical submucosal 2 or deeper cancer detected by non-ME and ME. This benefit probably occurs because patients diagnosed with clinical submucosal 2 or deeper cancer by non-ME and ME have no risk of an increase in overdiagnosis.

In this study, the accuracy of non-ME vs non-ME and ME for differentiating between pathological submucosal 2 or deeper and pathological submucosal 1 or shallower cancer was 74.0% vs 72.9%, respectively. These results suggest that cancer invasion depth may be misdiagnosed in 26.0% and 27.1% of patients with T1 ESCC, respectively. Considering the insufficient diagnostic performance, using endoscopic resection to confirm cancer invasion depth by pathological evaluation would be a reasonable strategy for patients with clinical T1 ESCC.^4^ If the cancer invasion depth is pathologically confirmed as mucosal cancer without vascular invasion, there is no need for additional treatment^4^; however, if histologic results indicate pathological submucosal 2 cancer, favorable survival can be expected by adding appropriate treatments, such as chemoradiotherapy^4^ or surgery.

### Limitations

This study has limitations. First, we evaluated the diagnostic performance of EUS longitudinally after the receipt of non-ME and ME rather than directly comparing EUS with non-ME and ME, and we were therefore unable to compare the performances of non-ME and ME vs EUS. However, EUS is usually conducted after non-ME and ME in daily clinical practice; therefore, the diagnostic performance of EUS after non-ME and ME is more relevant than direct comparison with non-ME and ME. In addition, the EUS examiners were not blinded to the non-ME and ME results because they needed these results to accurately evaluate the additional diagnostic performance of EUS.

Second, regarding the diagnostic category of cancer invasion depth, we evaluated the diagnostic accuracy of the methods for differentiating between pathological submucosal 1 or shallower and pathological submucosal 2 or deeper cancers. Although differentiation of pathological mucosal vs pathological submucosal cancers would be more clinically useful because this difference is directly used to select treatment, it was not possible to conduct the study with the aim of differentiating pathological mucosal and pathological submucosal cancers because there is currently no classification for differentiating these cancers based on non-ME and ME results.

Third, 31 of 300 patients (10.3%) who participated in the second registration were not included in the final analysis. However, given that this difference primarily occurred because patients chose to receive chemoradiotherapy rather than continue to participate in the diagnostic process, the difference might not have had substantial consequences for the results.

Fourth, we excluded clinical epithelial/lamina propria cancers; thus, the results only apply to clinical muscularis mucosal/submucosal cancers. However, given that EUS is rarely used for the diagnosis of clinical epithelial/lamina propria cancers, the exclusion of patients with clinical epithelial/lamina propria cancer was considered to be a reasonable decision.

Fifth, ME is not a standard procedure worldwide, which may have limited the generalizability of the study’s findings. However, the similar diagnostic performance of non-ME vs non-ME and ME with regard to sensitivity, specificity, and accuracy suggested that ME had minimal consequences for diagnosis, and the generalizability of the results was therefore unlikely to be altered by the use of ME. Sixth, although the experience criteria for endoscopists comprised 10 procedures for EUS and 20 procedures for ME, which did not ensure expert-level experience, these criteria were adequate to exclude novice endoscopists.

## Conclusions

The addition of EUS was not associated with improvements in the diagnostic performance of non-ME and ME for differentiating between pathological submucosal 1 or shallower and pathological submucosal 2 or deeper cancers. These results do not support the routine use of EUS after non-ME and ME for the evaluation of cancer invasion depth to determine whether endoscopic resection is indicated for patients with T1 ESCC.

## Notes

Supplement. **eTable 1.** Reasons for Exclusion **eTable 2.** Parameters for Differentiating ≥SM2 From ≤SM1 Cancers **eFigure.** Clinical Diagnosis, Treatments, and Pathological Diagnosis
