# Supplementary material for: Assessment of the Diagnostic Performance of Endoscopic Ultrasonography After Conventional Endoscopy for the Evaluation of Esophageal Squamous Cell Carcinoma Invasion Depth
Source: JAMA Netw Open. 2021 Sep 15;4(9):e2125317. doi: 10.1001/jamanetworkopen.2021.25317 (PMC8444025; doi:10.1001/jamanetworkopen.2021.25317)
Supplement: Supplement. — eTable 1. Reasons for Exclusion eTable 2. Parameters for Differentiating ≥SM2 From ≤SM1 Cancers eFigure. Clinical Diagnosis, Treatments, and Pathological Diagnosis [file jamanetwopen-e2125317-s001.pdf]

Supplementary Online Content

Ishihara R, Mizusawa J, Kushima R, et al. Assessment of the diagnostic performance of endoscopic ultrasonography after conventional endoscopy for the evaluation of esophageal squamous cell carcinoma invasion depth. *JAMA Netw Open*. 2021;4(9):e2125317.
doi:10.1001/jamanetworkopen.2021.25317

eTable 1. Reasons for Exclusion

eTable 2. Parameters for Differentiating ≥SM2 From ≤SM1 Cancers

eFigure. Clinical Diagnosis, Treatments, and Pathological Diagnosis

This supplementary material has been provided by the authors to give readers additional information about their work.

© 2021 Ishihara R et al. *JAMA Network Open*.

eTable 1. Reasons for Exclusion

Excluded before the second registration	No (%)
Cancer invasion depth of cEP, cLPM, or cT2 on endoscopic diagnosis by non-ME+ME	33 (45.8)
Could not receive endoscopic examination within 14 days after the first registration	11 (15.3)
Major axis length > 50 mm	10 (13.9)
Diagnoses of metastasis	6 (8.3)
Cancers located in the abdominal esophagus	4 (5.6)
Other reason	8 (11.1)
Excluded from the final analyses	No (%)
Did not undergo cancer resection	19 (61.3)
Diagnoses of metastasis	2 (6.5)
Histology other than squamous cell carcinoma	1 (3.2)
Improper second registration (e.g. second registration after EUS)	9 (29.0)

EP: epithelium, LPM: lamina propria, non–ME: non-magnifying endoscopy, ME: non-magnifying endoscopy, EUS: endoscopic ultrasonography.

© 2021 Ishihara R et al. *JAMA Network Open*.

eTable 2. Parameters for Differentiating ≥SM2 From ≤SM1 Cancers

	Sensitivity No. (% [95%CI])	Specificity No. (% [95%CI])	PPV No. (% [95%CI])	NPV No. (% [95%CI])	Accuracy No. (% [95%CI])
Non-ME	60/115 (52.2% [42.7%-61.6%])	139/154 (90.3% [84.4-94.5])	60/75 (80.0% [69.2-88.4])	139/194 (71.6% [64.8-77.9])	199/269 (74.0% [68.3-79.1])
Non-ME+ME	58/115 (50.4% [41.0%–59.9%])	138/154 (89.6% [83.7%–93.9%])	58/74 (78.4% [67.3-87.1])	138/195 (70.8% [63.8-77.0])	196/269 (72.9% [67.1%–78.1%])
Non-ME+ME +EUS	74/115 (64.3% [54.9%–73.1%])	125/154 (81.2% [74.1%–87.0%])	74/103 (71.8% [62.1-80.3])	125/166 (75.3% [68.0-81.7])	199/269 (74.0% [68.3%–79.1%])

CI: confidence interval, SM: submucosa, non–ME: non-magnifying endoscopy, ME: non-magnifying endoscopy, EUS: endoscopic ultrasonography, PPV: positive predictive value, NPV: negative predictive value

© 2021 Ishihara R et al. *JAMA Network Open*.

eFigure. Clinical Diagnosis, Treatments, and Pathological Diagnosis

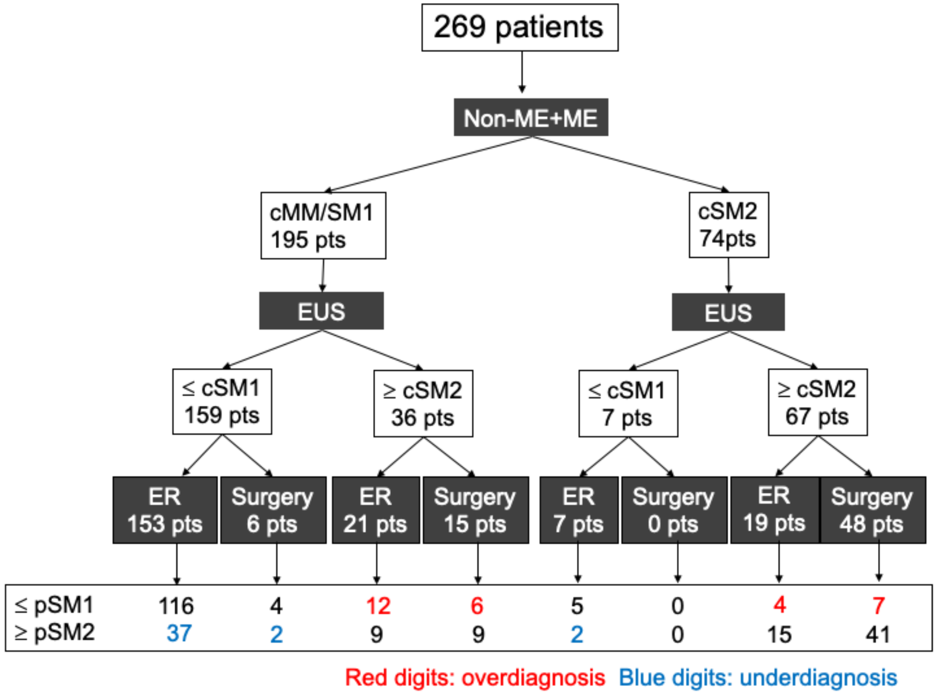

Non–ME: non-magnifying endoscopy, ME: non-magnifying endoscopy, EUS: endoscopic ultrasonography, SM: submucosa, ER: endoscopic resection, pts: patients.

© 2021 Ishihara R et al. *JAMA Network Open*.
